# Determining DNA–Protein Binding Affinities and Specificities from Crude Lysates Using a Combined SILAC/TMT Labeling Strategy

**DOI:** 10.1021/acs.jproteome.3c00248

**Published:** 2023-07-19

**Authors:** Cathrin Gräwe, Miguel Hernandez-Quiles, Pascal W. T. C. Jansen, Annika Brimmers, Michiel Vermeulen

**Affiliations:** † Department of Molecular Biology, Faculty of Science, Radboud Institute for Molecular Life Sciences, Oncode Institute, Radboud University Nijmegen, 6525 GA Nijmegen, The Netherlands; ‡ Division of Molecular Genetics, The Netherlands Cancer Institute, 1066CX Amsterdam, the Netherlands

**Keywords:** protein−DNA interaction, binding specificity, binding affinity, DNA pull-down, TMT, SILAC, higher-order multiplexing

## Abstract

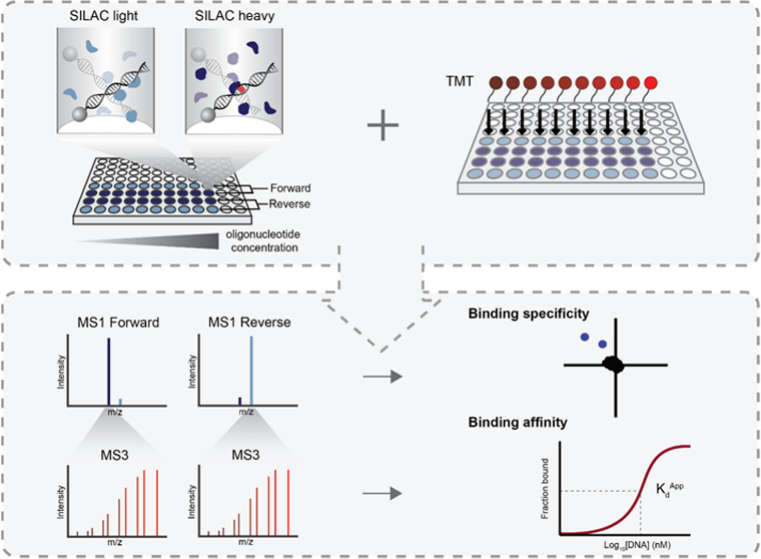

In recent years, quantitative mass spectrometry-based interaction proteomics technology has proven very useful in identifying specific DNA–protein interactions using single pull-downs from crude lysates. Here, we applied a SILAC/TMT-based higher-order multiplexing approach to develop an interaction proteomics workflow called Protein–nucleic acid Affinity and Specificity quantification by MAss spectrometry in Nuclear extracts or PASMAN. In PASMAN, DNA pull-downs using a concentration range of specific and control DNA baits are performed in SILAC-labeled nuclear extracts. MS^1^-based quantification to determine specific DNA–protein interactions is then combined with sequential TMT-based quantification of fragmented SILAC peptides, allowing the generation of Hill-like curves and determination of apparent binding affinities. We benchmarked PASMAN using the SP/KLF motif and further applied it to gain insights into two CGCG-containing consensus DNA motifs. These motifs are recognized by two BEN domain-containing proteins, BANP and BEND3, which we find to interact with these motifs with distinct affinities. Finally, we profiled the BEND3 proximal proteome, revealing the NuRD complex as the major BEND3 proximal protein complex in vivo. In summary, PASMAN represents, to our knowledge, the first higher-order multiplexing-based interaction proteomics method that can be used to decipher specific DNA–protein interactions and their apparent affinities in various biological and pathological contexts.

## Introduction

DNA–protein interactions are essential for the regulation of gene expression. Transcription factors interact with specific DNA sequences that are present in regulatory regions in the genome. Upon binding, a variety of other proteins are recruited, which eventually results in either activation or repression of transcription.^1,2^ Various technologies are continuously applied and developed to study DNA–protein interactions. This involves mainly next-generation sequencing-based approaches, such as ChIP sequencing, bisulfite sequencing, or ATAC sequencing, which provide information about the localization of transcription factors on DNA. Furthermore, these techniques have given us a more profound understanding how the DNA sequence, DNA modifications, or genome accessibility affect transcription factor binding.^3^ In contrast, mass spectrometry-based techniques provide information from a protein-centric point of view and can identify proteins that bind to a specific DNA sequence or modification of interest.^4,5^ Protein- and DNA-centric methods are complementary, each providing a unique set of information, which together contributes to the understanding of gene expression regulation. For example, the TCTCGCGAGA motif (hereafter referred to as the CGCG motif) was previously identified as a strong activating DNA motif;^6−8^ however, transcription factors that bind this motif to activate target genes remained elusive. Recently, DNA pull-downs combined with quantitative mass spectrometry identified BANP as a specific and high-affinity binder of the CGCG motif. Genomic sequencing-based experiments further showed that BANP regulates chromatin accessibility, thereby allowing the activation of direct target genes.^9^ This illustrates the complementary nature of genomic- and proteomics-based approaches to studying transcription factor biology.

As shown above, DNA pull-downs combined with quantitative mass spectrometry facilitate the identification of specific interactors for a particular DNA sequence of interest. However, to fully understand DNA–protein interactions, it is crucial to not only determine binding specificity (i.e., whether binding occurs) but also binding affinity (i.e., how strong this binding is). Affinity quantification is often overlooked when studying DNA–protein interactions, in particular for workflows in which DNA pull-downs are coupled to mass spectrometry. To address this gap, we recently developed a new method, called Protein–nucleic acid Affinity Quantification by MAss spectrometry in Nuclear extracts or PAQMAN, that enables us to determine apparent binding affinities between proteins and DNA sequences of interest in the context of crude nuclear extracts.^10,11^ In PAQMAN, a concentration range of immobilized DNA is incubated with a fixed amount of nuclear extract. After incubation and washes, bound proteins are digested and labeled with isobaric 10-plex tandem mass tags (TMT). Finally, labeled peptides from the ten DNA pull-downs are combined and analyzed in a single LC-MS run. Apparent dissociation constants (*K*_d_^App^s), a measure of binding affinity, can be calculated by determining the DNA concentration at which 50% of maximum protein binding is observed. Using this workflow, apparent binding affinities in the range of ∼1 to 600 nM can be calculated for multiple proteins interacting with a DNA sequence of interest in a single experiment. However, binding specificity information is not obtained using the PAQMAN workflow.

Here, we developed Protein–nucleic acid Affinity and Specificity quantification by MAss spectrometry in Nuclear extracts or PASMAN. In PASMAN, conventional DNA pull-downs coupled to SILAC-based quantification to determine binding specificity is integrated with TMT-based quantification of fragment ions to determine binding affinities, a strategy that is known as higher-order multiplexing. We benchmarked PASMAN using the SP/KLF motif and further applied the workflow to characterize DNA binding for two related BEN domain-containing proteins, BANP and BEND3. PASMAN is, to our knowledge, the first higher-order multiplexing workflow for interaction proteomics and represents a valuable addition to the available toolbox to study DNA–protein interactions in a proteome-wide, unbiased manner.

## Materials and Methods

### Cell Culture, SILAC Labeling, and Cell Lysate Preparation

K562 cells were cultured in RPMI 1640 (Thermo) supplemented with 10% FBS (Gibco) and 1% penicillin–streptomycin (Thermo). HeLa Kyoto cells were cultured in DMEM (Thermo) supplemented with 10% FBS and 1% penicillin–streptomycin. SILAC labeling was performed by culturing HeLa cells in SILAC DMEM (88420; Thermo) supplemented with 10% dialyzed FBS (DS-1003; Dundeecell), 1× glutamax (35050-061; Thermo), 1× penicillin–streptomycin, 36.5 mg/mL l-lysine (light/K0, Sigma, L8662; or heavy/K8, Silantes, 211604102), and 16.8 mg/mL arginine (light/R0, Sigma, A6969; or heavy/R10, Silantes, 201604102). Cells were cultured in SILAC medium for two weeks, after which heavy amino acid incorporation was checked. Only when heavy amino acid incorporation was >95% were cells expanded for nuclear extract preparation. Cells were regularly tested for mycoplasma contamination.

Crude nuclear extracts were prepared as described previously.^11^ Briefly, cells were harvested using trypsin (Promega), and the obtained cell pellet was washed twice with PBS. The cell pellet was resuspended in 5 volumes of buffer A (10 mM HEPES KOH pH 7.9, 15 mM MgCl2, 10 mM KCl) and incubated for 10 min on ice. Cells were pelleted by centrifugation (400*g*, 5 min, 4 °C) and resuspended in 2 volumes of buffer A supplemented with 0.15% NP-40 and EDTA-free complete protease inhibitor (CPI). Then, cells were lysed by dounce homogenization, and crude nuclei were collected by centrifugation (3200*g*, 15 min, 4 °C). After washing with PBS, crude nuclei were resuspended in buffer C (420 mM NaCl, 20 mM HEPES pH 7.9, 20% (v/v) glycerol, 2 mM MgCl2, 0.2 mM EDTA, 0.1% NP-40, CPI, and 0.5 mM DTT), incubated for 90 min while rotating at 4 °C. Afterward, the nuclear lysate was centrifuged (20,000*g*, 30 min, 4 °C) and the soluble nuclear fraction was collected. Obtained nuclear extracts were aliquoted, snap-frozen in liquid nitrogen, and stored at −80 °C.

Whole-cell lysates were prepared by harvesting cells using trypsin, followed by resuspension of the cell pellet in 5 volumes of RIPA buffer (150 mM NaCl, 50 mM Tris pH 8, 1 mM EDTA, 10% glycerol, 1% NP-40, 1 mM DTT, CPI). After 60 min of rotating at 4 °C, lysates were spun down at 20,000 g for 30 min, and the supernatant was collected. The cleared lysate was snap-frozen in liquid nitrogen and stored at −80 °C.

### Plasmid Constructs and Transfection

Plasmid constructs were custom ordered from TwistBiosciences. The human BEND3 coding sequence was fused to V5-miniTurbo and cloned into the pTwist CMV overexpression plasmid. HeLa cells were transfected using X-tremeGENE 9 (ROCHE). For one 10 cm dish, 15 μL of X-tremeGENE reagent was diluted in 500 μL of Opti-MEM (Thermo) and, together with 5 μg of plasmid DNA, incubated at room temperature for 15 min. This mixture was added dropwise to the cells. The medium was replaced with fresh medium the next day.

### DNA Pull-Downs

DNA oligonucleotides were ordered from Integrated DNA Technologies (IDT) with 5′-biotinylation of the forward strand. The sequences of the oligonucleotides used can be found in Table 1. To anneal oligonucleotides, the forward strand was combined with 1.5× molar excess of the reverse strand in annealing buffer (10 mM Tris pH 8.0, 50 mM NaCl, 1 mM EDTA) and heated to 95 °C for 10 min, followed by slowly cooling them to room temperature.

**Table 1:** DNA Oligonucleotide Baits Used in This Study^a^

name	sequence forward (5′->3′)	Sequence reverse (5′->3′)
SP/KLF bait	/5Biosg/GAGAG**CCCCGCCCC**CTGGCT	AGCCAGGGGGCGGGGCTCTC
SP/KLF negative control bait	/5Biosg/GAGAGAAAATAAAACTGGCT	AGCCAGTTTTATTTTCTCTC
CGCG bait	/5Biosg/GCCGCCGCCCT**TCTCGCGAGA**CTGCCGGGCC	GGCCCGGCAGTCTCGCGAGAAGGGCGGCGGC
CGCG negative control bait	/5Biosg/GCCGCCGCCCTTCGGCAAGTCCTGCCGGGCC	GGCCCGGCAGGACTTGCCGAAGGGCGGCGGC
methylated CGCG bait	/5Biosg/GCCGCCGCCCT**TCT/iMe-dC/G/iMe-dC/GAGA**CTGCCGGGCC	GGCCCGGCAGTCT/iMe-dC/G/iMe-dC/GAGAAGGGCGGCGGC
BEND3 bait	/5Biosg/GCCGCCGCCCT**CCCACGCG**CTGCCGGGCC	GGCCCGGCAGCGCGTGGGAGGGCGGCGGC
BEND3 negative control bait	/5Biosg/GCCGCCGCCCTGCAGCCCCCTGCCGGGCC	GGCCCGGCAGGGGGCTGCAGGGCGGCGGC
methylated BEND3 bait	/5Biosg/GCCGCCGCCCT**CCCA/iMe-dC/G/iMe-dC/G**CTGCCGGGCC	GGCCCGGCAG/iMe-dC/G/iMe-dC/GTGGGAGGGCGGCGGC

a Bases defining the motif are marked in bold.

DNA pull-downs were performed in duplicate as described previously.^12^ In short, for each reaction, 20 μL of streptavidin–sepharose bead slurry (GE Healthcare) was prepared by washing twice with 1 mL of DNA binding buffer (DBB; 1 M NaCl, 10 mM Tris pH 8.0, 1 mM EDTA, 0.05% NP-40). Next, 500 pmol of annealed DNA oligonucleotides was immobilized on beads in a total volume of 600 μL DBB. After 30 min of rotating at 4 °C, beads were washed twice with DBB and once with protein incubation buffer (PIB; 150 mM NaCl, 50 mM Tris pH 8.0, CPI, 0.25% NP-40, 1 mM DTT). Per pull-down, 500 μg of nuclear extract was added in a total volume of 600 μL PIB and incubated by rotating for 90 min at 4 °C. Next, beads were washed three times with 1 mL of PIB and twice with 1 mL of PBS. After removing excess PBS with a syringe, samples were prepared for western blotting or mass spectrometry analysis (see sections below).

For western blotting, beads were resuspended in 50 μL of sample buffer (50 mM Tris pH 6.5, 100 mM DTT, 70 mM SDS, 1.5 mM bromophenol blue, 1.1 M glycerol) and boiled at 95 °C for 10 min. Samples were resolved on polyacrylamide gels and transferred to 0.22 μm nitrocellulose membranes. The membranes were blocked using 5% milk or BSA dissolved in PBS-T and subsequently probed with primary antibodies (BANP: abcam, ab72076; BEND3: abcam, ab220896; GAPDH: Santa Cruz Biotechnology, sc-32233; SP1: Sigma; PARP1: #9542; Streptavidin-HRP-conjugated: Thermo Fisher Scientific, PA1-26848). Afterward, membranes were washed three times with PBS-T and incubated with the corresponding HRP-conjugated secondary antibody (Dako). Before imaging, membranes were washed three times with PBS-T and once with PBS. Membranes were imaged with an ImageQuantTM LAS-4000 (GE Healthcare) using the Pierce ECL Western Blotting Substrate (Thermo Fisher Scientific). All raw western blot images are shown in Supporting Figure 2.

For mass spectrometry, beads were resuspended in 50 μL of elution buffer (2 M urea, 100 mM Tris pH 8.5, 10 mM DTT) and incubated for 20 min while shaking at room temperature. Proteins were alkylated using 50 mM iodoacetamide (IAA), followed by 10 min incubation while shaking in the dark. Next, proteins were digested on-bead by adding 0.25 μg of trypsin and incubated while shaking for 2 h at room temperature. Afterward, samples were spun down, and the supernatant was collected. Beads were washed once more with 50 μL of elution buffer, and the supernatant was collected and added to the previously collected supernatant. Proteins were continued to be digested overnight with an additional 0.1 μg of trypsin. The next day, samples were purified using StageTips. Dimethyl labeling on StageTips was done as described previously.^13^

### PAQMAN and PASMAN

PAQMAN and PASMAN experiments were mainly performed as described previously.^10,11^ DNA oligonucleotides were annealed as described above. Every PAQMAN experiment was performed in duplicate, each experiment consisting of ten individual DNA pull-downs with different DNA oligonucleotide concentrations. A dilution series was prepared consisting of ten DNA oligonucleotide concentrations (ranging from 0.15 nM to 3 μM) in DBB. DNA pull-downs were performed in a 96-well filter plate (Millipore, MSBVS1210). The wells were first washed once with 70% ethanol (v/v) and twice with DBB. Then, per reaction, 20 μL of streptavidin–sepharose bead slurry was added and washed twice with DBB. Afterward, 150 μL of oligonucleotides of each titration point of the prepared dilution series was added to the beads and incubated overnight and shaken at 4 °C. The next morning, every well was washed once with DBB and twice with PIB. Per pull-down, 100 μg of the nuclear extract or SILAC-labeled nuclear extract was added to the corresponding pull-downs and incubated for 2 h and shaken at 4 °C. For PASMAN, a label-swap experiment was performed for each replicate. Then, wells were washed six times with washing buffer (150 mM NaCl, 100 mM TEAB). Samples were prepared for western blot analysis as described above or for mass spectrometry analysis. For mass spectrometry analysis, beads were resuspended in 50 μL of elution buffer (20% (v/v) methanol, 80 mM TEAB, 10 mM TCEP) and incubated and shaken for 30 min at room temperature. Proteins were reduced with 50 mM IAA for 10 min in the dark. On-bead digestion was performed by adding 0.25 μg of trypsin, followed by incubation for 2 h and shaking at room temperature. Samples were collected into a collection plate by centrifugation (500*g*, 5 min), and wells were flushed again with an additional 50 μL of elution buffer, which was collected into the same collection plate. Proteins were allowed to digest further overnight.

The next day, the sample volume was reduced to 10 μL by vacuum centrifugation. Each 0.8 mg of the TMT 10-plex labeling reagent (Thermo Fisher Scientific) was resuspended in 101 μL of anhydrous acetonitrile. To each sample, 10 μL of the corresponding TMT label was added and incubated while shaking for 1 h at room temperature in the dark. Afterward, labeling reactions were quenched by adding 10 μL of 1 M Tris pH 8.0 and incubating while shaking for 30 min at room temperature. All ten pull-down samples of each PAQMAN replicate were combined. For SILAC-labeled PASMAN experiments, the respective light- and heavy-labeled PAQMAN pairs were combined as well. Samples were acidified with trifluoroacetic acid and desalted by StageTipping.^13^

### Proximity-Labeling Experiments

For proximity-labeling experiments using miniTurbo, cells were treated with 50 μM biotin (B20656, Invitrogen) for 10 min preharvesting. Cells were harvested 48–72 h post-transfection and whole-cell lysates were prepared as described above. Western blotting was done to validate the biotinylation activity of our construct.

Pull-down experiments of biotinylated proteins were performed in triplicate with biotin-treated HeLa wild-type and transfected cells. For each pull-down, 2 mg of whole-cell lysate was combined with 20 μL of streptavidin–sepharose bead slurry, RIPA buffer, and 2 μL of ethium bromide in a total volume of 600 μL. The samples were incubated for 90 min while rotating at 4 °C. Afterward, beads were washed three times with RIPA buffer and twice with PBS. On-bead digestion and sample preparation for mass spectrometry analysis were performed as described above.

### Mass Spectrometry Analysis

Samples were eluted from StageTips with buffer B (80% acetonitrile, 0.1% formic acid), concentrated to 5 μL by SpeedVac centrifugation, and resuspended to a final volume of 12 μL in buffer A (0.1% formic acid). Peptides were separated by liquid chromatography using an Easy-nLC 1000 system (Thermo Fisher Scientific). For this, 5 μL of the sample was loaded onto a 30 cm column (heated to 40 °C) packed in-house with 1.8 μm Reprosil-Pur C18-AQ (Dr Maisch). For PASMAN samples, 2 μL were loaded onto the column.

For PAQMAN and PASMAN experiments, peptides were eluted from the column using a gradient from 7–15% buffer B over 5 min, from 15–35% buffer B over 214 min, from 35–50% buffer B over 5 min, and from 50–95% buffer B over 1 min, followed by 5 min hold at 95% buffer B. Mass spectrometry analysis was performed on a Thermo Fusion Tribrid instrument using the built-in method Thermo synchronous precursor selection (SPS) MS3. The detailed acquisition method was described previously.^11^

All other pull-downs were eluted from the column using a gradient from 12–30% buffer B over 43 min, from 30–60% buffer B over 10 min, and from 60–95% buffer B over 1 min. Mass spectrometry analysis was performed on a Thermo Exploris 480 instrument. The mass spectrometer was operated in top20 data-dependent acquisition mode. Target values for full MS were set at the 3e6 AGC target and a maximum injection time of 20 ms. Full MS^1^ spectra were recorded at a resolution of 120,000 over a scan range of 350–1300 m/z. Target values for MS^2^ were set at the 7.5e5 normalized AGC target with a maximum injection time of 22 ms. The isolation width was set to 1.6 *m*/*z* and the intensity threshold to 5e3. For fragmentation, the HCD collision energy was set at 28%. MS^2^ spectra were recorded at a resolution of 150,000 with an isolation width of 1.6. Peptides with a charge state of 2–6 were included for MS/MS analysis.

### Mass Spectrometry Data Analysis

All raw mass spectrometry spectra were processed using ProteomeDiscoverer 3.0 (Thermo Fisher Scientific) and searched against the UniProt curated human proteome database released in June 2017. Identified proteins were filtered for common contaminants. All data visualization was done with Python 3.

Dimethyl-labeled samples were analyzed using a modified workflow that is based on the built-in dimethylation 3plex method. For quantification, light-dimethyl-labeled peptides (+28.031 Da) and heavy-dimethyl-labeled peptides (+32.056 Da) were used. Low abundance resampling was enabled. Only proteins that were quantified in all 4 channels were used for downstream analysis. Outlier statistics were used to identify significant proteins. Proteins were considered significant with one interquartile range for both forward and reverse experiments.

For label-free samples, the built-in LFQ workflow was used with standard settings. Downstream analysis was performed with Perseus (version 2.0.6.0)^14^ using the normalized and scaled abundances calculated by ProteomeDiscoverer. Only proteins that were identified with at least 2 peptides, of which had 1 to be unique, were considered for further analysis. Proteins had to be identified and quantified in 3/3 replicates in at least one condition. Missing values were imputed from a normal distribution. A two-sample t-test was performed to identify proteins that are significantly enriched.

For SILAC-labeled samples, the built-in SILAC quantification workflow was used with standard settings. Arg10 and Lys8 were set as variable modifications for samples that were labeled either light or heavy. For PASMAN analysis, a new quantification method was added in ProteomeDiscoverer that is based on the built-in SILAC 2plex method. For the light-labeled channel, TMT6plex was added as the modification on lysines and N-termini. For the heavy-labeled channel, Arg10 and K8-TMT6plex (237.177 Da; H(20) C(2) 13C(10) N(-1) 15N(3) O(2)) were added as side-chain modifications and TMT6plex was added on N-termini. Obtained results were exported and further analyzed using Python. Proteins that have no unique peptides or that could not be quantified in all four channels were filtered out. Outlier statistics were used to identify significant proteins. Proteins were considered significant with one interquartile range for both forward and reverse experiments.

PAQMAN and PASMAN data were essentially analyzed as described earlier.^11^ The built-in TMT 10-plex quantification workflow was used with standard settings. For heavy SILAC-labeled peptides, a new quantification method was added that is based on the built-in TMT 10-plex quantification method. K8-TMT6plex (237.177 Da) was added as residue modification, and TMT6plex was kept as an N-terminal modification. In addition, Arg10 was set as a static modification in the processing workflow. Obtained results were exported, and apparent binding affinities were determined by using an in-house Python script.

TMT labeling efficiency was determined by essentially analyzing raw mass spectrometry files as described above. However, the TMT 6-plex reagent mass was set as a dynamic modification on lysines and the peptide N-terminus. Labeling efficiency was calculated as described previously using the Peptide Groups output file.^11^ Only when labeling of peptides was successful were data used for further analysis (Supporting Figure 1).

## Results

### Experimental Setup

During the last two decades, various quantification strategies have been developed for mass spectrometry-based proteomics. These strategies can broadly be divided into methods that quantify proteins at the intact peptide or the MS^1^ level or at the fragment ion or the MS^2/3^ level.^15^ To further increase multiplexing, peptide and fragment ion-based quantification can also be combined. This strategy is commonly referred to as higher-order multiplexing^16^ and has been applied to address various biological questions.^17−22^

**Figure 1:**
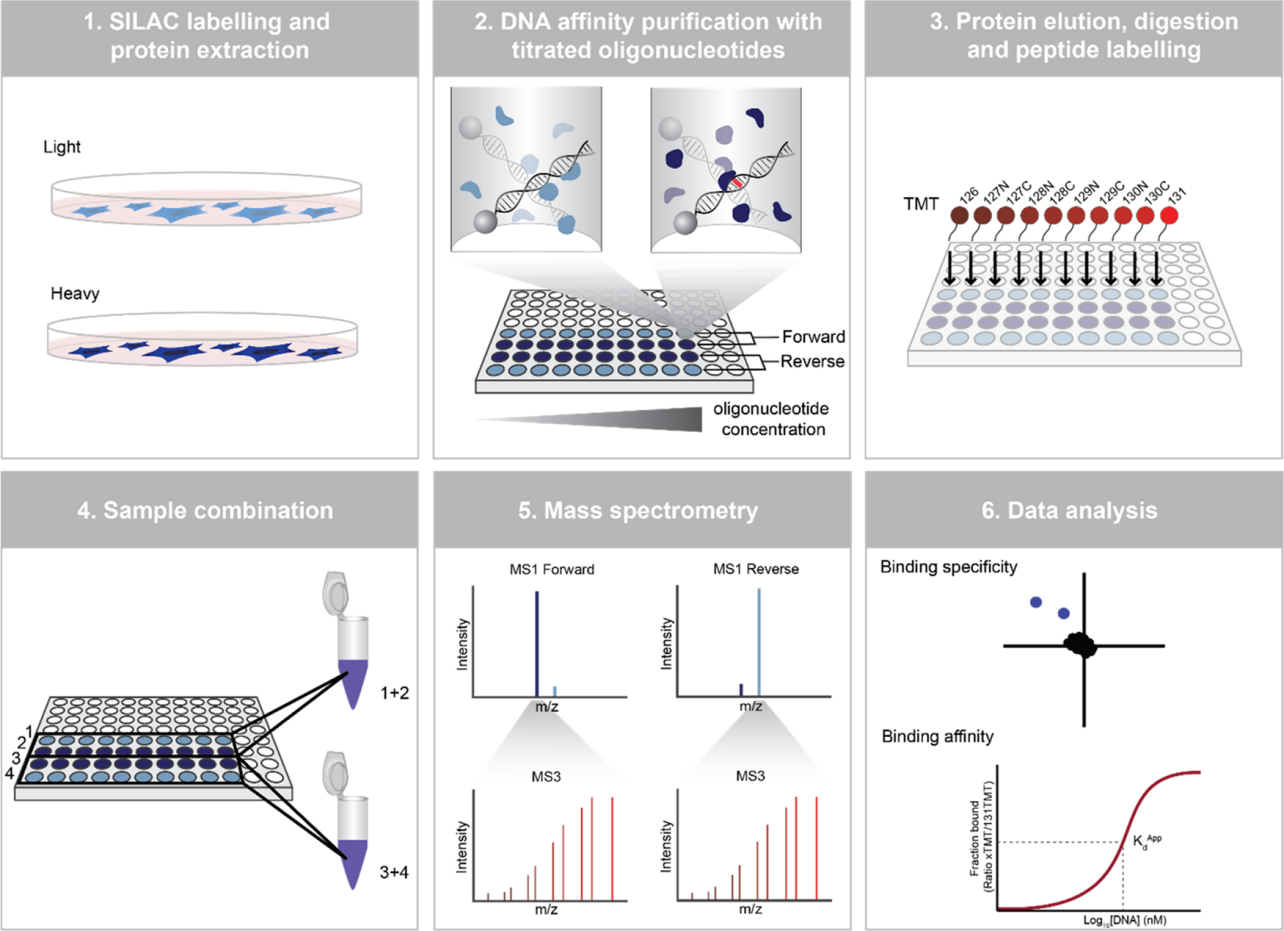
Schematic overview of the experimental setup to determine DNA–protein binding specificity and affinity in a single experiment. First, nuclear extracts from SILAC-labeled cells are prepared. DNA baits containing the motif of interest or a negative control motif are designed with 5′ biotinylation of the forward strand. A series with ten 3-fold dilutions of these DNA baits is prepared and immobilized in a 96-well filter plate. SILAC-labeled nuclear extracts are added to the DNA baits and incubated for 90 min. After extensive washes, bound proteins are digested, followed by 10-plex TMT labeling. Samples of a titration series, as well as samples of the forward or reverse experiment, are combined. Peptides are identified and quantified by mass spectrometry analysis. Obtained SILAC data are used to identify proteins that bind specifically to the motif of interest, whereas TMT data are used to generate Hill-like curves for *K*_d_^App^ determination.

Here, we set out to broaden the application of higher-order multiplexing in the context of DNA pull-downs with the aim of determining protein–DNA binding specificity and affinity in a single experiment. The experimental setup is shown in Figure 1. DNA baits with a 5′ biotin tag on the forward strand and either containing a motif of interest or a negative control bait are designed. The negative control contains a scrambled version of the motif of interest placed into the same flanking sequence. As in PAQMAN experiments, DNA baits are prepared in a concentration range of ten 3-fold dilutions (from 0.15 nM to 3 μM) and then immobilized on streptavidin beads in a 96-well filter plate. Next, 100 μg of the “light” SILAC-labeled nuclear extract is added to the concentration range of DNA baits containing the motif of interest, whereas the “heavy” labeled nuclear extract is added to the negative control baits. Furthermore, a label-swap experiment is performed as a replicate measurement. Following incubation, nonbound proteins are washed away, after which bound proteins are digested with trypsin. Obtained peptides are then labeled with TMT. All of the TMT-labeled “light” samples are then combined with all of the TMT-labeled “heavy” samples and the final sample (derived from 20 DNA pull-downs in total) is analyzed by LC-MS. Consequently, 40 DNA pull-downs are analyzed in total in two LC-MS runs. Binding specificity is determined by calculating SILAC ratios of peptides bound to either the motif of interest or the control motif. Binding affinities are determined as in PAQMAN experiments; Hill-like curves are generated from quantified TMT reporter ions, from which apparent dissociation constants (*K*_d_^App^s) are calculated (the lower the *K*_d_^App^ value, the stronger the binding). We call this method Protein–nucleic acid Affinity and Specificity quantification by MAss spectrometry in Nuclear extracts or PASMAN.

### Benchmarking Using the SP/KLF Motif

**Figure 2:**
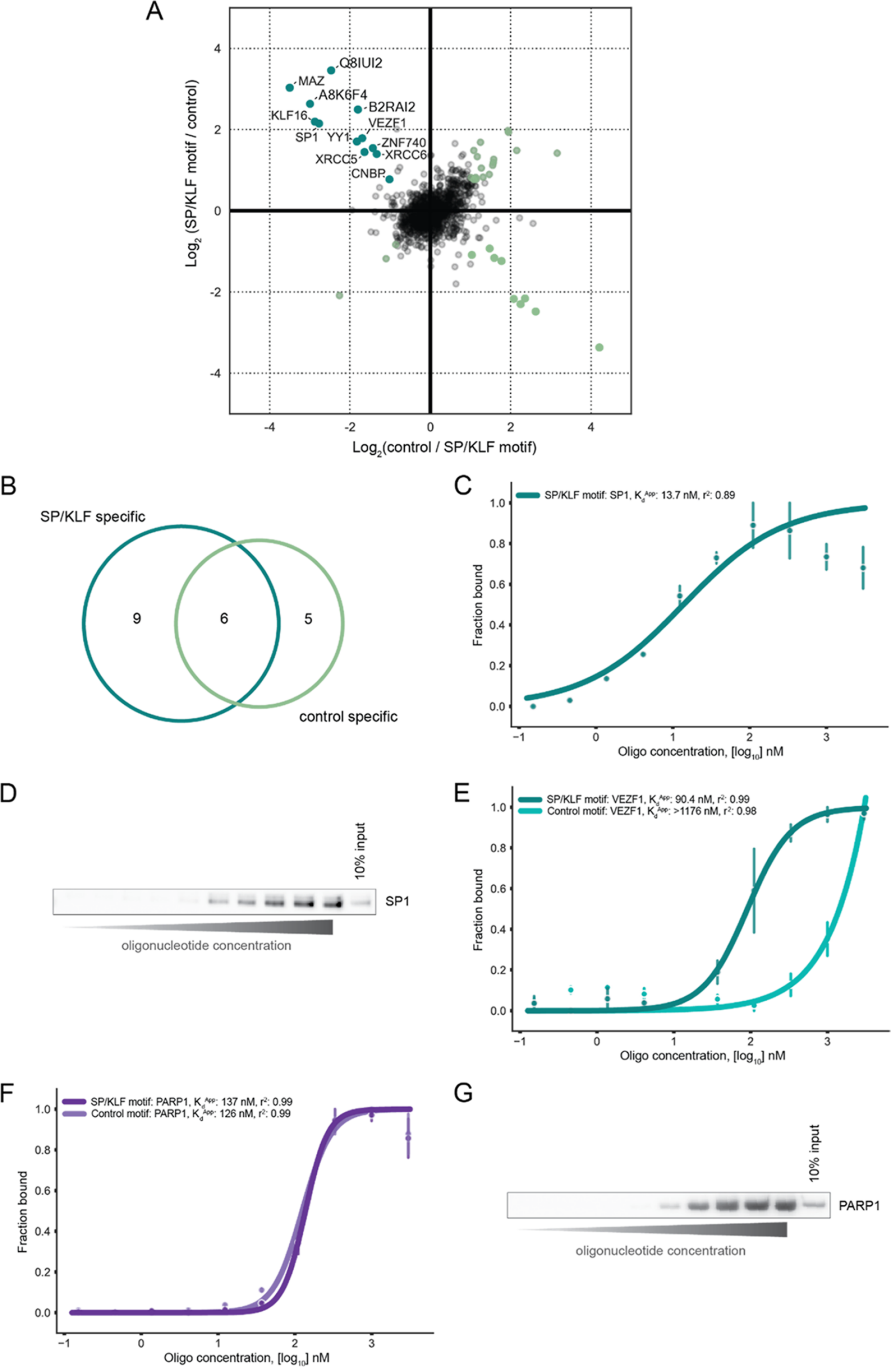
Benchmarking of PASMAN using the SP/KLF motif. (A) SILAC Log_2_ ratios of the forward and reverse experiments are plotted against each other. Outlier statistics with IQR (interquartile range) cutoff of 1 was applied to call significant proteins. Significant binders for the SP/KLF motif are colored teal, and all other significant proteins are colored green. (B) Venn diagram showing the number of proteins for which a *K*_d_^App^ could be calculated and how many proteins overlap when comparing binders of the SP/KLF motif with the control motif. (C) Hill-like curve for SP1 binding to the SP/KLF motif obtained by PASMAN. Data points represent the mean of two experiments, and standard errors are the standard error of the mean. (D) PAQMAN, followed by western blotting, to validate SP1 binding to the SP/KLF motif. (E) Hill-like curve for VEZF1 binding to SP/KLF and the exponential curve for VEZF1 binding to the negative control motif obtained by PASMAN. (F) Hill-like curve for PARP1 binding to SP/KLF and the negative control motif obtained by PASMAN. (G) PAQMAN, followed by western blotting, to validate PARP1 binding to the SP/KLF bait.

To benchmark PASMAN, we applied the workflow to the SP/KLF consensus motif.^10,23^ As expected, we identified SP1 and a number of other proteins as specific interaction partners of the SP/KLF motif compared to the negative control bait (Figure 2A, Supporting Figure 1A). When comparing the number of specific binders with previous experiments, it is apparent that PASMAN identifies fewer SP/KLF motif interactors. This is probably due to the low amount of nuclear extract used per pull-down and the increased sample complexity due to higher-order multiplexing (also see the Discussion section). Still, using TMT-based quantification of fragmented SILAC peptides, we were able to determine a *K*_d_^App^ in the range of ∼ 6–600 nM for 20 proteins (Figure 2B), binding to either the SP/KLF motif, including SP1 (Figure 2C,D), the negative control bait, or both. For VEZF1, another motif-specific interactor, we were able to determine a binding affinity for the SP/KLF motif (*K*_d_^App^ 90.4 nM), whereas a lower affinity was observed for the negative control bait, which we estimated to be a minimal of *K*_d_^App^ >1176 nM by fitting an exponential curve (Figure 2E). Some proteins, such as PARP1, have a high affinity for both baits (Figure 2F,G). Some of these proteins represent DNA repair proteins that have a high affinity for short DNA stretches but do not display sequence-specific binding. For most other proteins, we were only able to determine a *K*_d_^App^ for either the motif of interest or the control sequence, indicating that these proteins display strong DNA sequence binding specificity. We noticed that some data points that were used for the generation of Hill-like curves show a decrease in signal at the highest titration points. To further investigate this, we performed PAQMAN experiments, followed by western blot analysis, which did not reveal a reduction of SP1 and PARP1 at higher bait concentrations (Figure 2D,G). These data suggest that a decrease in TMT signals at the highest bait concentration is likely technical in nature. In summary, this experiment serves as proof of principle that PASMAN can be used to determine DNA–protein binding specificity and affinity in a single workflow by applying higher-order multiplexing.

### Benchmarking Using the CGCG Motif

**Figure 3:**
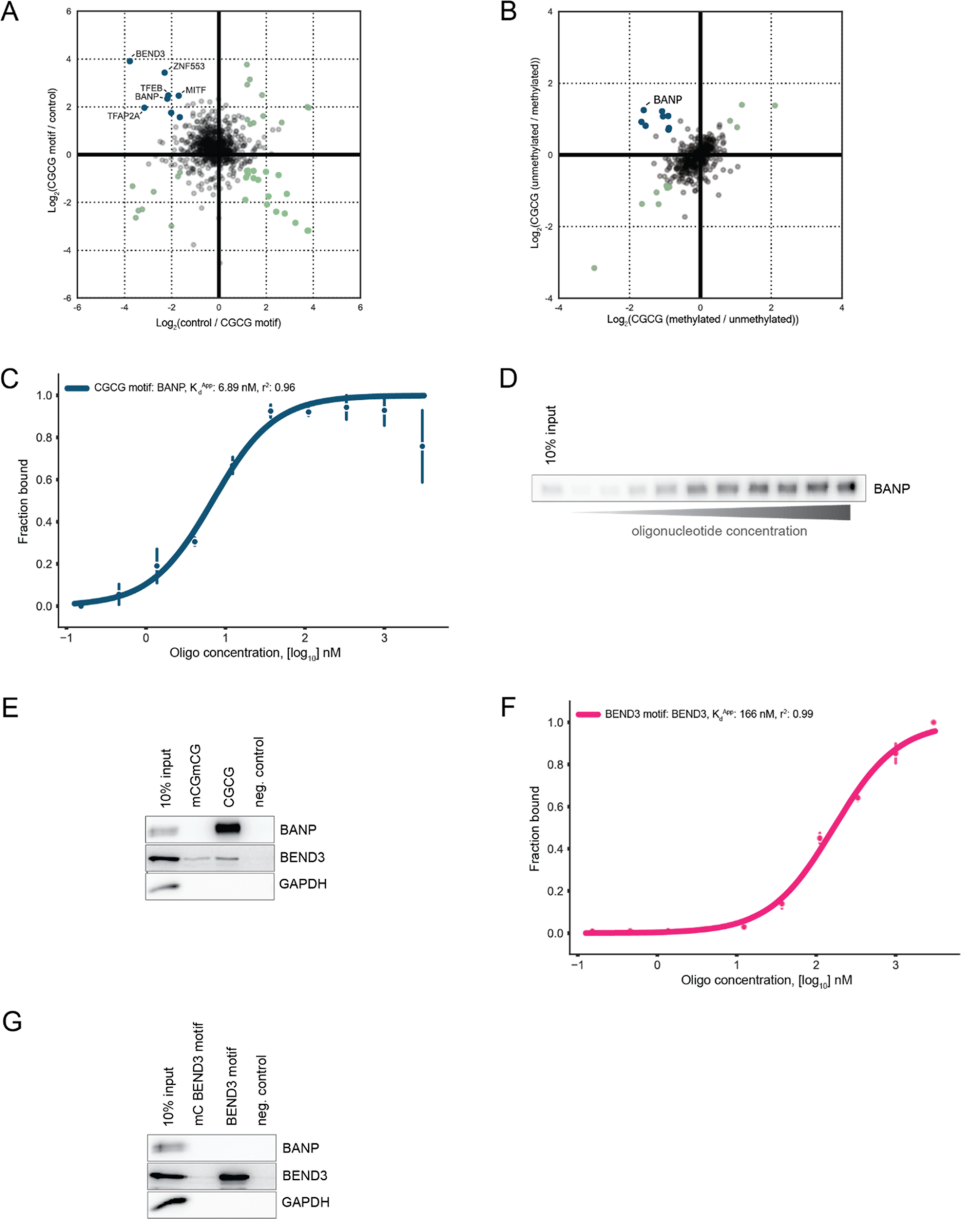
BANP and BEND3 interact with distinct motifs. (A) Standard DNA pull-down, followed by dimethyl labeling using the CGCG motif. The experiment was done in duplicate with a label swap of replicates. Significant proteins are labeled teal for the CGCG motif, and all other significant proteins are labeled green. (B) SILAC Log_2_ ratios are plotted against each other for PASMAN profiling the CGCG motif. (C) Hill-like curve for BANP binding to the CGCG motif using TMT data obtained by PASMAN. (D) PAQMAN, followed by western blotting, to validate BANP binding to the CGCG motif. (E) Western blot analysis of BANP and BEND3 binding to the CGCG motif. (F) Hill-like curve for BEND3 binding to the BEND3 motif obtained from a standard PAQMAN experiment. (G) Western blot analysis of BANP and BEND3 binding to the BEND3 motif.

Next, we applied PASMAN to the CGCG motif, for which the BEN domain-containing protein BANP was recently identified as a specific and high-affinity binder. We first performed standard DNA pull-down experiments using the human HeLa nuclear extract, and as expected, BANP specifically binds to the CGCG motif (Figure 3A). Furthermore, we performed a PAQMAN experiment for the motif in the human K562 nuclear extract, which confirmed a high-affinity binding of BANP to the CGCG motif in human cells (*K*_d_^App^ 11.2 nM; Supporting Table 2). We then applied PASMAN to the CGCG motif in SILAC-labeled HeLa nuclear extracts. MS^1^-based quantification of the SILAC data revealed BANP as a specific binder for the unmethylated CGCG motif compared to a methylated version of the CGCG motif (Figure 3B). MS^3^-based fragmentation of the SILAC-labeled peptides and subsequent quantification of the TMT reporter ions revealed a *K*_d_^App^ of 6.89 nM between BANP and the CGCG motif (Figure 3C,D), which is very similar to measurements obtained using PAQMAN (Supporting Table 2).^9^ However, as observed for the SP/KLF motif, we noticed that the amount of identified specific interactors for the CGCG motif is lower using our PASMAN workflow compared to standard DNA pull-downs. As previously mentioned, this may be due to technical issues associated with the multiplexed labeling strategy (also see the Discussion section).

Interestingly, BEND3 was also identified as a specific interactor of the CGCG motif in HeLa nuclear extract when performing a standard DNA pull-down (Figure 3A). BEND3, like BANP, is a BEN domain-containing protein and has previously been linked to major satellites and bivalent genes.^24,25^ Further validation of our mass spectrometry data by western blot analysis confirmed that BEND3 specifically binds to the CGCG motif; however, these data suggest that this binding is much weaker compared to BANP. Moreover, similar to BANP, the binding of BEND3 to the CGCG motif seems to be methylation-sensitive (Figure 3E). A recent study identified a binding motif for BEND3 in mouse ESCs.^25^ We, therefore, performed a PAQMAN experiment with this motif (CCCACGCGC) in HeLa nuclear extract, which revealed that BEND3 interacts with this motif with a relatively low affinity (*K*_d_^App^ 166 nM) (Figure 3F) compared to typical transcription factor-DNA motif affinities. Additionally, DNA pull-downs, followed by western blot analysis, confirmed that BEND3 interacts more strongly with its motif compared to the BANP CGCG motif and that this interaction is methylation-sensitive (Figure 3G), as reported previously.^24−26^ In summary, these experiments illustrate the added value of combined affinity and specificity measurements compared to specificity measurements alone since BANP and BEND3 both specifically interact with the CGCG motif, but affinity measurements revealed that their binding affinity for the motif differs by the order of magnitude.

### BEND3 Interactome

**Figure 4:**
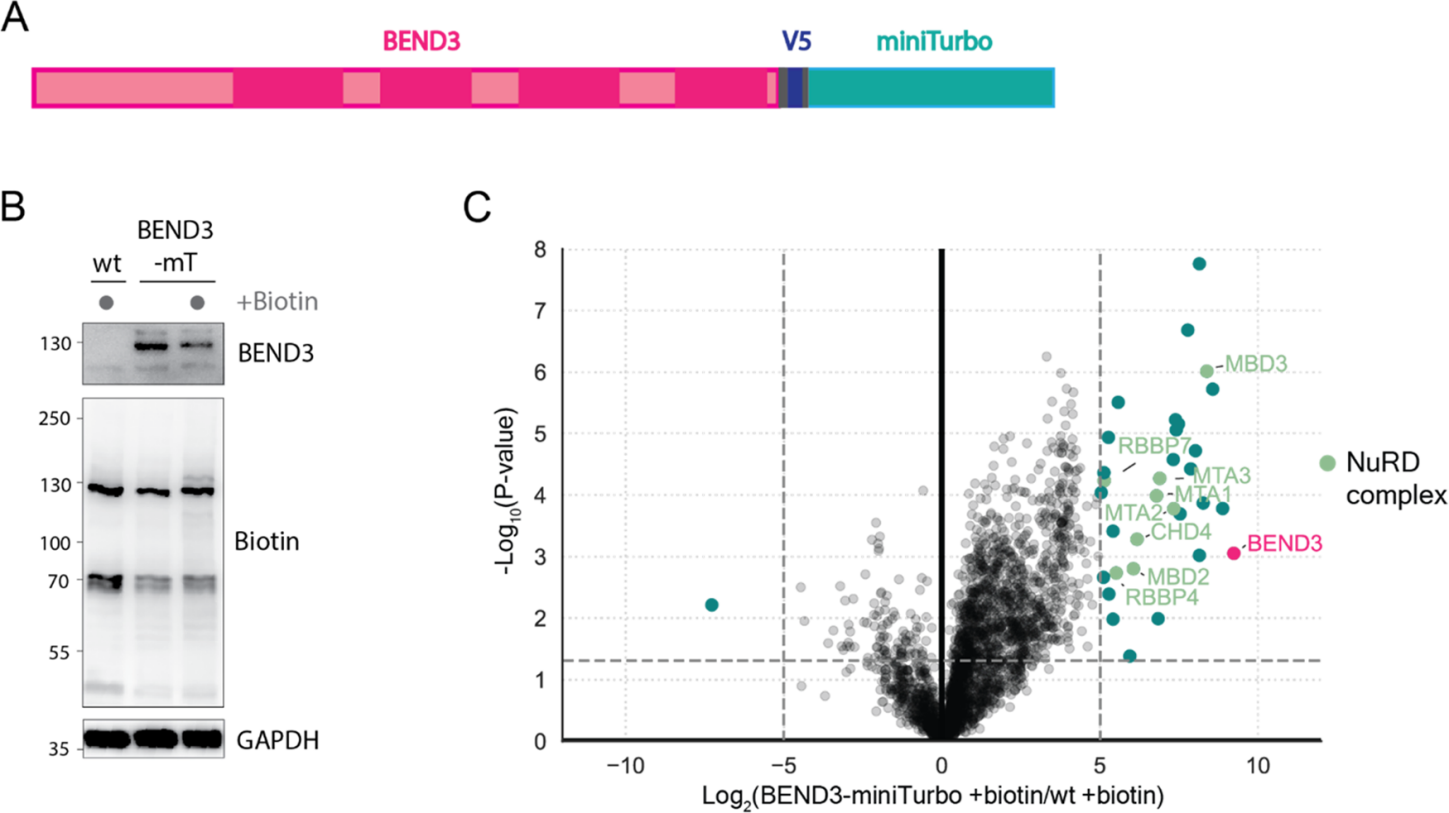
Identification of potential BEND3 interaction partners. (A) Design of the BEND3-V5-miniTurbo overexpression construct. (B) Western blot analysis of biotinylation activity of the BEND3-V5-miniTurbo overexpression construct in HeLa cells. (C) Mass spectrometry analysis of proteins captured by the biotin pull-down. Label-free quantitation of triplicates was used, and proteins were called significant with a *p*-value of <0.05 and a Log_2_ fold-change of 5. Significant proteins are colored teal, significant proteins belonging to the NuRD complex are colored green, and the bait is colored magenta.

Previous studies have shown that BEND3 is involved in the repression of transcription,^24,25,27^ while BANP was identified as a potent activator of transcription.^9^ To further explore the possible function of BEND3, we performed proximity biotinylation experiments in HeLa cells using a BEND3-miniTurbo fusion construct (Figure 4A). We first confirmed that this fusion protein shows biotinylation activity in HeLa cells (Figure 4B). We then performed streptavidin-based pull-downs in wild-type or BEND3-miniTurbo extracts following biotin labeling. Bound proteins were eluted and analyzed by LC-MS. Raw mass spectrometry data were analyzed using the standard built-in ProteomeDiscoverer workflow and also using the recently developed CHIMERYS search algorithm (MSAID). We identified significantly more proteins using the CHIMERYS algorithm (3966 vs 5880 proteins) (Supporting Table 1), demonstrating the added value of artificial intelligence in boosting identification rates of classic DDA mass spectrometry data. The application of CHIMERYS, however, did not result in the identification of more specific outliers in this specific experiment. Mass spectrometry-based analysis revealed that subunits of the NuRD complex are in close proximity to BEND3 in vivo (Figure 4C). This result confirms that the biological function of BEND3 may be opposed to BANP. Whereas BANP acts as a potent activator of transcription, BEND3 seems to be associated with repression and/or fine-tuning of gene expression. Further studies are required to precisely understand the molecular function of BEND3.

## Discussion

Here, we developed PASMAN, which represents, to the best of our knowledge, the first interaction proteomics workflow applying higher-order multiplexed quantification to determine DNA–protein interaction specificity and affinity in a single workflow. When comparing PASMAN to a regular DNA pull-down workflow, we noticed that we typically identify fewer significant outliers. This is probably due to the increased sample complexity that is observed when performing DNA pull-downs using different bait concentrations, ranging from low nM to low μM measured in a single LC-MS run. Furthermore, the use of MS^3^ scans in PASMAN requires relatively long duty cycles, which compromises sequencing depth.^28,29^ TMT reporter ions can also be quantified at the MS^2^ level, but we decided to use MS^3^-based fragmentation and quantification due to the well-described higher ratio distortion/compression caused by co-isolation and co-fragmentation of peptides using MS^2^-TMT quantification.^30−32^ We anticipate that further hardware and software developments (for example, artificial intelligence-based analysis tools such as CHIMERYS) to acquire and analyze mass spectrometry data including higher-order multiplexed proteomics data will improve PASMAN data quality and sequencing depth. Furthermore, the continuous development of quantification approaches and their multiplexing capacity will improve protein identification rates (e.g., increasing TMT multiplexing capacity).^31^ Another reason for the observed low number of significant outliers in PASMAN experiments may be the fact that only 100 μg of the input nuclear extract is used for each DNA pull-down. This reduced amount of input is necessary to avoid overloading the LC column (20 DNA pull-downs are analyzed in a single LC-MS run), but the reduced protein concentration for each DNA pull-down may also result in lower peptide ratios between the specific and control pull-down at the MS^1^ level. Furthermore, at several used bait concentrations (from low to nM to low μM), there may not be an observed difference in binding specificity between the specific and control baits, which may lower the overall protein ratio observed at the MS^1^ level.

In this manuscript, we show that BEND3 interacts weakly with a CGCG-containing motif, for which BANP was identified as a high-affinity interactor. Recently, a consensus BEND3 binding motif was identified.^25^ Interestingly, PAQMAN revealed that BEND3 interacts with this motif with a *K*_d_^App^ of 166 nM, which is a relatively low affinity compared to typical interactions between transcription factors and their preferred motif. Indeed, this affinity is significantly lower compared to the affinity between BANP and its preferred CGCG-containing motif, which is ∼15 nM.^9^ We were intrigued by the fact that BEND3 is mainly known to be associated with the repression of gene expression, while it was recently shown that BANP is involved in the activation of gene expression.^33^ Previous studies suggest that BEND3 associates with repressive chromatin complexes, including NuRD.^24,25,34−36^ We set out to identify interaction partners of BEND3 by proximity labeling, which revealed NuRD complex subunits as in vivo BEND3 proximal proteins, further supporting a potential link between BEND3 and NuRD.^24,34,37,38^ Despite this co-localization on chromatin, BEND3 does not impact the genome-wide localization of NuRD but regulates PRC2 recruitment to chromatin.^24,25^ The exact mechanisms underlying these interesting observations remain elusive and further work is needed to unravel the complex relationship between BEND3, NuRD, and Polycomb. BEN domain-containing proteins are evolutionally conserved (the human genome encodes for 9 BEN domain-containing proteins), and although some of these proteins are important for chromatin biology, others are completely uncharacterized.^39,40^ We anticipate that important insights into cellular functioning will be obtained by studying this relatively uncharacterized protein family.

In vivo binding of transcription factors across the genome is not only regulated by the DNA sequence but also by other factors such as chromatin accessibility, the availability of co-factors, and epigenetic modifications. These factors are typically not taken into consideration in DNA pull-down experiments from crude lysates. However, the recently reported identification of BANP as the long sought-after transcription factor that interacts with the CGCG motif in CpG islands clearly demonstrates that in vitro approaches form a strong basis to identify DNA–protein interactions that are relevant in vivo.^9^ Furthermore, PAQMAN and PASMAN workflows are not limited to DNA–protein interactions but can also be extended to identify proteins interacting with modified nucleosomes, secondary DNA structures, or post-translational modifications, as we have shown previously.^10,41^ Interaction proteomics workflows such as PAQMAN/PASMAN that are used to determine binding affinities between proteins and a single DNA motif of interest are complementary to workflows that determine binding affinities of a single protein across the entire genome, such as the recently developed method BANC-seq.^42^ Altogether, the integration of interaction proteomics and genomic methods provide a powerful systems toolbox to characterize interactions between transcription factors and the genome in a truly quantitative manner, which is essential to generate gene regulatory networks that can accurately model gene expression programs and cell fate in health and disease.

## Data Availability

All raw mass spectrometry data have been deposited at the ProteomeXchange Consortium via the PRIDE partner repository with the data set identifier PXD041674.

## Supporting Information Available

Table containing results of all mass spectrometry experiments performed in this study (XLSX)
Table containing *K*_d_^App^s determined in this study (XLSX)
TMT labeling efficiency of SILAC-labeled peptides in PASMAN for the SP/KLF motif; example MS spectra for SP1; and raw western blot images (PDF)
