# Supplementary material for: Determining DNA–Protein Binding Affinities and Specificities from Crude Lysates Using a Combined SILAC/TMT Labeling Strategy
Source: J Proteome Res. 2023 Jul 19;22(8):2683–93. doi: 10.1021/acs.jproteome.3c00248 (PMC10407929; doi:10.1021/acs.jproteome.3c00248)
Supplement: Supplementary file 3 — pr3c00248_si_003.pdf [file pr3c00248_si_003.pdf]

Supporting Information

Determining DNA-protein binding affinities and specificities from crude lysates using a combined SILAC/TMT labeling strategy

Cathrin Gräwe[1], Miguel Hernandez-Quiles[1,2], Pascal W. T. C. Jansen[1], Annika Brimmers[1], Michiel Vermeulen[1,2]*

[1]Department of Molecular Biology, Faculty of Science, Radboud Institute for Molecular Life Sciences, Oncode Institute, Radboud University Nijmegen, 6525 GA Nijmegen, The Netherlands.

[2]Division of Molecular Genetics, The Netherlands Cancer Institute, 1066 CX Amsterdam, the Netherlands.

* corresponding author: michiel.vermeulen@ru.nl

CONTENT:

The supporting information includes: Supplemental Figures S1 and S2, and supplemental Tables S1 and S2, which are provided as excel files.

Figure S1 shows the TMT labelling efficiency of SILAC labeled peptides in PASMAN for the SP/KLF motif. Furthermore, example MS spectra for SP1 are shown.

Figure S2 shows raw western blot images used in this study.

Table S1 summarizes results of all mass spectrometry experiments performed in this study.

Table S2 summarizes apparent binding affinities measured in this study.

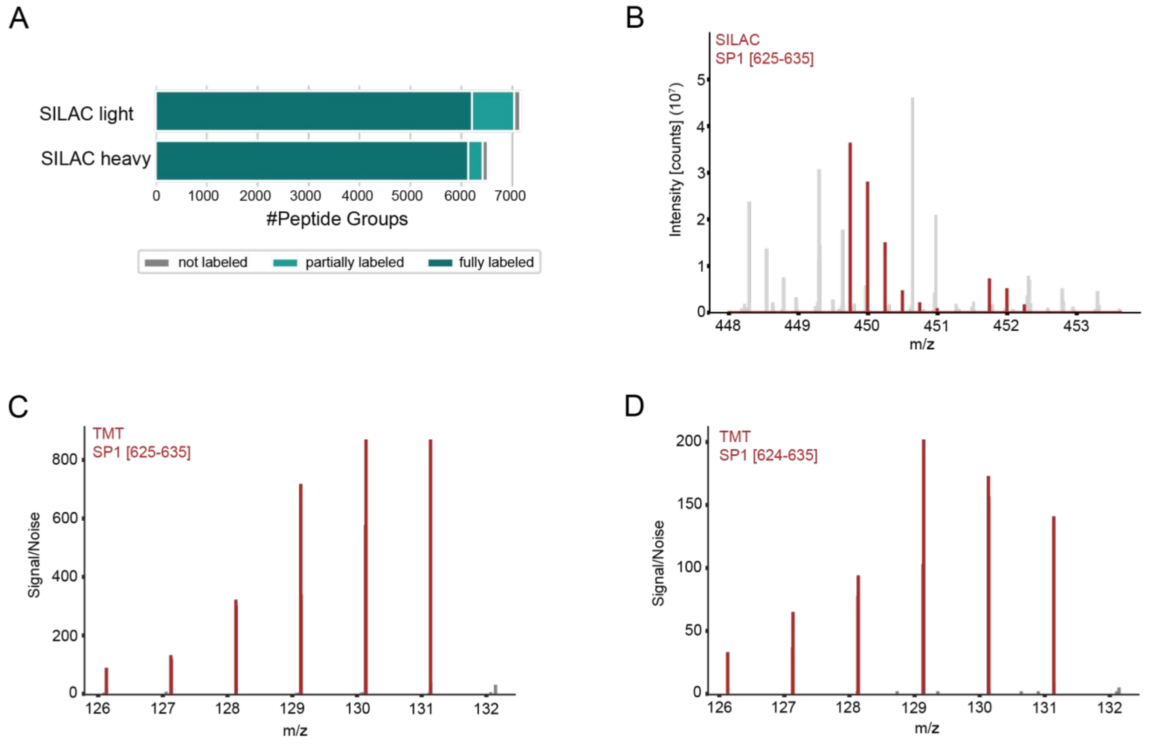

Supplemental Figure 1: TMT labelling efficiency and example PASMAN spectra used for quantification for the SP/KLF motif. (A) TMT labelling efficiency of SILAC labelled peptides. (B) Example of an SILAC MS1 spectrum of an SP1 precursor ion. (C) Example MS3 spectrum of a “light” SILAC precursor ion of a quantified SP1 peptide. (D) Example MS3 spectrum of a “heavy” SILAC precursor ion of a quantified SP1 peptide.

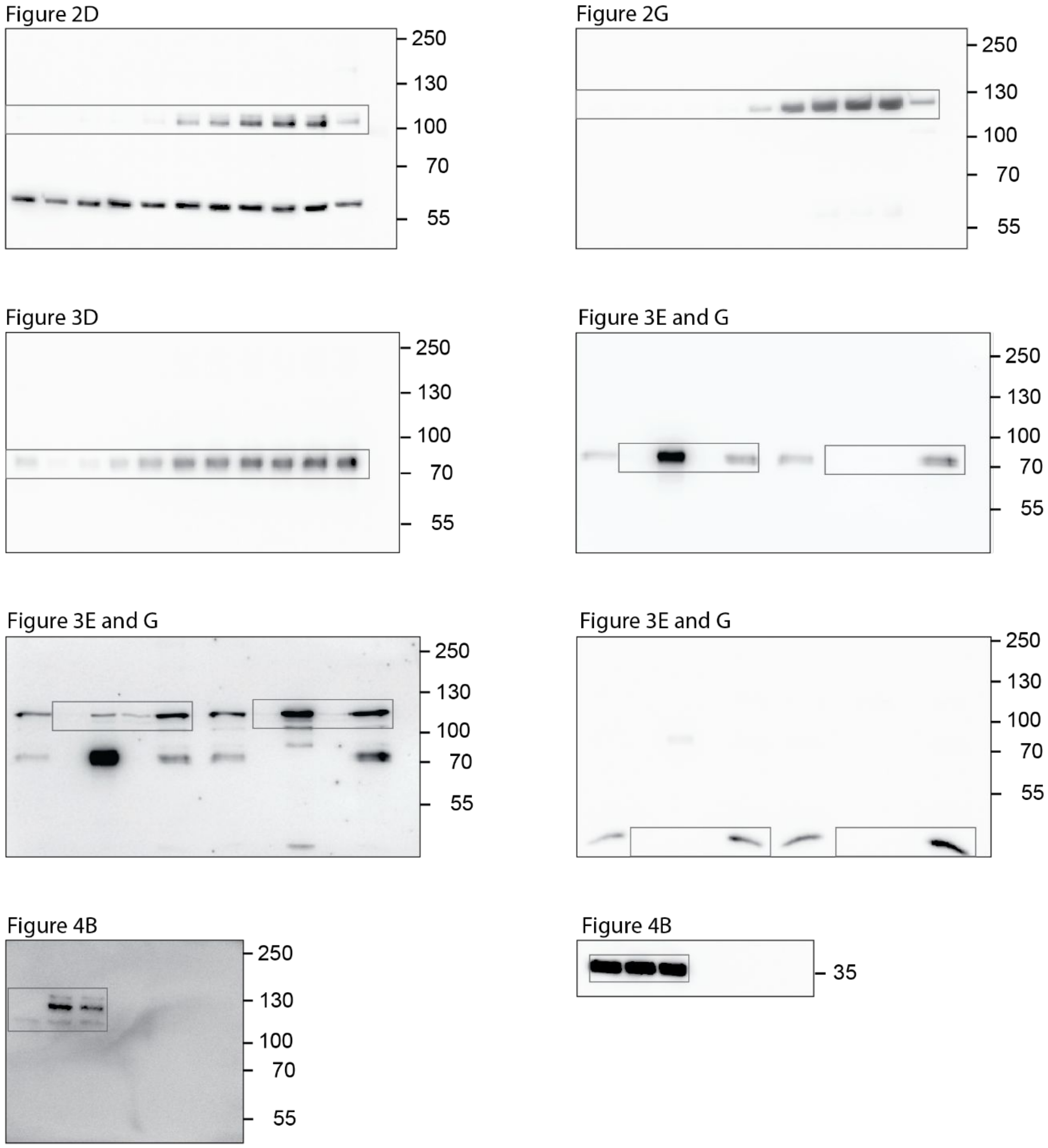

Supplemental Figure 2: Raw images for western blot analysis shown in this study. Each raw image is related to the figure indicated above each blot. Cropped lanes are indicated by gray boxes.
